# Short- and long-term exposure to trace metal(loid)s from the production of ferromanganese alloys by personal sampling and biomarkers

**DOI:** 10.1007/s10653-022-01218-8

**Published:** 2022-02-22

**Authors:** B. Markiv, L. Ruiz-Azcona, A. Expósito, M. Santibáñez, I. Fernández-Olmo

**Affiliations:** 1 Departamento de Ingenierías Química y Biomolecular, Universidad de Cantabria, Santander, Spain; 2 Departamento de Enfermería, Universidad de Cantabria, Santander, Spain

**Keywords:** Biomarkers, Manganese, Trace metal(loid)s, Ferromanganese alloy production, Environmental exposure

## Abstract

The environmental exposure to trace metal(loid)s (As, Cd, Cu, Fe, Mn, Pb, and Zn) was assessed near a ferromanganese alloy plant using filters from personal particulate matter (PM) samplers (bioaccessible and non-bioaccessible fine and coarse fractions) and whole blood as short-term exposure markers, and scalp hair and fingernails as long-term biomarkers, collected from volunteers (*n* = 130) living in Santander Bay (northern Spain). Bioaccessible and non-bioaccessible metal(loid) concentrations in coarse and fine PM from personal samplers were determined by ICP-MS after extraction/digestion. Metal(loid) concentration in biomarkers was measured after alkaline dilution (whole blood) and acid digestion (fingernails and scalp hair) by ICP-MS as well. Results were discussed in terms of exposure, considering the distance to the main Mn source, and sex. In terms of exposure, significant differences were found for Mn in all the studied fractions of PM, As in whole blood, Mn and Cu in scalp hair and Mn and Pb in fingernails, with all concentrations being higher for those living closer to the Mn source, with the exception of Cu in scalp hair. Furthermore, the analysis of the correlation between Mn levels in the studied biomarkers and the wind-weighted distance to the main source of Mn allows us to conclude that scalp hair and mainly fingernails are appropriate biomarkers of long-term airborne Mn exposure. This was also confirmed by the significant positive correlations between scalp hair Mn and bioaccessible Mn in coarse and fine fractions, and between fingernails Mn and all PM fractions. This implies that people living closer to a ferromanganese alloy plant are exposed to higher levels of airborne metal(loid)s, mainly Mn, leading to higher levels of this metal in scalp hair and fingernails, which according to the literature, might affect some neurological outcomes. According to sex, significant differences were observed for Fe, Cu and Pb in whole blood, with higher concentrations of Fe and Pb in males, and higher levels of Cu in females; and for Mn, Cu, Zn, Cd and Pb in scalp hair, with higher concentrations in males for all metal(loid)s except Cu.

**Supplementary Information:**

The online version contains supplementary material available at 10.1007/s10653-022-01218-8.

## Introduction

Exposure to elevated levels of metal(loid)s that are present in the environment is concerning due to the resulting adverse health effects (carcinogenic, neurotoxic, etc.), even if some of them can be considered as essential, required at trace levels for metabolism, such as copper (Cu), iron (Fe), manganese (Mn) or zinc (Zn) (Maret, 2016; Zoroddu et al., 2019). Three main routes of exposure to metal(loid)s are known: (i) ingestion from foods and water; (ii) dermal contact; and (iii) inhalation.

The inhalation route of exposure is particularly important for people living close to airborne metal(loid)s emission sources in urban and industrial areas (Bauer et al., 2020), such as metallurgy, steelworks, combustion (including transportation) and incineration. Airborne metal(loid)s are bound to particulate matter (PM), its particle size being a key parameter on the fate of such pollutants in the human body (Kelly & Fussell, 2012). Thus, the thoracic fraction (equivalent to PM_10_) includes the tracheobronchial fraction (i.e. inhaled particles that penetrate beyond the larynx but do not reach the non-ciliated airways) and the respirable fraction (i.e. inhaled particles that enter the non-ciliated airways). Particles of the 2.5–10 µm size fraction are mostly deposited in the pharyngeal and tracheal region (i.e. constitute the tracheobronchial fraction), but they can be swallowed, reaching the gastrointestinal tract, where they come into contact with gastric juice. Smaller particles (i.e. the respirable fraction) can travel deeper into the alveolar region of the lungs, where they interact with the lung fluid; the interstitial lung fluid has a neutral pH; however, these small particles can be phagocytosed by alveolar macrophages, resulting in a more acidic medium (pH 4.5). Dissolved metal(loid)s can then reach the circulatory system (Expósito et al., 2021; Mukhtar & Limbeck, 2013).

A simple marker of the exposure to metal(loid)s by inhalation route is the distance between the known source(s) and the receptors; for example, previous studies have used the distance between the participants’ home in cross-sectional epidemiological studies and the source of emission of certain air pollutants as a preliminary indicator of exposure (Vimercati et al., 2016; Zubero et al., 2010). The importance of being downwind or upwind of the emission source has been considered by Haynes et al. (2012), calculating the wind direction-weighted distance for short-term exposure, addressing Mn exposure in the vicinity of a Mn alloy plant by children living in the town of Marietta (Ohio) in the USA.

The assessment of the exposure to metal(loid)s by the inhalation route can be done directly by measuring or modelling their levels in ambient air, or indirectly by measuring their concentration in selected biomarkers. Stationary samplers have been widely used in extensive PM sampling campaigns for the analysis of metal(loid)s concentration in filters, providing information about the long-term exposure to such pollutants, but with the limitation of obtaining information only from specific sites where these samplers were located (Fulk et al., 2016). This limitation is addressed using personal PM samplers, which are easy to wear by exposed individuals, with the added advantage of accounting for changes in exposure during short sampling periods. Filters collected from these samplers can be analysed for total metal(loid)s content or for the bioaccessible concentration, which reflects the amount of each pollutant to be solubilised by a human synthetic fluid.

The degree of inhalation exposure can also be determined by the analysis of specific exposure biomarkers (Fernández-Olmo et al., 2021). However, the levels of metal(loid)s in such biomarkers can also account for the other routes of exposure, mainly the ingestion route; this is especially true for essential trace elements, which are included in the usual diet, but can sometimes also apply to non-essential ones, such as arsenic (As), cadmium (Cd) or lead (Pb). The usefulness of an exposure biomarker is assessed for its ability to characterise and differentiate exposed and non-exposed groups, as well as for their ability to predict health disorders, anticipating any deterioration of health (Viana et al., 2014; Zheng et al., 2011). Although there is no current consensus on which biomarker best defines the dose–effect relationship, and furthermore, the use of these biomarkers does not differentiate how much of these metal(loid)s enter the body by inhalation, biomarkers of exposure have been used in epidemiological studies designed to assess the exposure to some metal(loid)s near airborne metal(loid)s sources (Haynes et al., 2015; Rodrigues et al., 2018; Viana et al., 2014).

Blood has been considered as a short-term exposure biomarker to some metal(loid)s (Freire et al., 2015; Stojsavljević et al., 2019); it was frequently used to assess exposure to non-essential metals such as Pb and Cd (Henríquez-Hernández et al., 2017; Wong & Lye, 2008), highlighting the possibility of assessing acute intoxications due to the efficiency and sensitivity of the use of this biomarker. However, there are doubts about its use to assess exposure to essential trace elements such as Mn, Fe, Cu or Zn, due to their homeostatic regulation. For example, the mean-life time of Mn in blood is much shorter than in other tissues and cellular compartments, so some authors consider that it is not a good biomarker of short-term exposure to Mn (Jiang et al., 2007; Kim et al., 2015), being this more noticeable in the case of inhalation exposure, since despite finding higher levels in the blood of occupationally exposed individuals, it is difficult to quantify how the pulmonary uptake of airborne Mn contributes to its increase in blood (Roth, 2006).

Other candidates have been considered for long-term exposure to metal(loid)s, such as hair and nails (Butler et al., 2019; Fernández-Olmo et al., 2021; Nakaona et al., 2020; Parhizkar et al., 2021), because of their slow growth: between 1 and 1.2 cm/month of hair (Van Neste & Rushton, 2016) and about 3.47 mm/month of fingernails (Yaemsiri et al., 2010). They are easy to sample, transport, handle and store (Haynes et al., 2015; Menezes-Filho et al., 2009; Sukumar & Subramanian, 2007), although it is not possible to discriminate between the specific exposure route to them, providing us with a final concentration resulting from inhalation, oral and dermal routes. When considering these biomarkers, it is necessary to be cautious about the use of cosmetics such as dyes or nail polishes. Although Directive 1223/2009 on cosmetic products forbids the use of heavy metals as additives in EU member states (European Parliament & Council of the European Union, 2009), a recent study has pointed out that coloured nail polish still contains some metal(loid)s (Ceballos et al., 2021).

Recent studies published by our research group highlighted the elevated levels of airborne Mn in Santander Bay (Cantabria region, Northern Spain), exceeding the WHO annual guideline (i.e. 150 ng/m^3^ of Mn) (Hernández-Pellón & Fernández-Olmo, 2019; Hernandez-Pellón et al., 2017). These elevated levels were mainly due to the emissions from a ferromanganese alloy production plant located in this area; approximately, 91% of air Mn emitted in this area comes from this factory (Otero-Pregigueiro et al., 2018). In addition, a source apportionment study carried out in this area also identified Cd, Fe, Pb and Zn as tracers from this plant (Hernández-Pellón & Fernández-Olmo, 2019). However, other local sources of metal(loid)s apart from the ferromanganese alloy exist, such as road traffic, combustion and other industrial sources, leading to moderate levels of Cu and As (Hernández-Pellón & Fernández-Olmo, 2019).

Therefore, this area was selected to assess the exposure to airborne Mn and other trace metal(loid)s in the healthy adult population living in the vicinity of this ferromanganese alloy plant. For this purpose, the following short-term exposure markers to such metal(loid)s were taken from 130 volunteers recruited in a cross-sectional study: 24-h personal PM samples of different particle sizes (PM_10-2.5_ and PM_2.5_), which accounted for the inhalation route of exposure, as well as whole blood samples. For long-term exposure, scalp hair and fingernails samples were collected, with the aim of finding associations between the amounts of metal(loid)s inhaled and those processed by the body, to determine the best biomarker of exposure to airborne Mn and other metal(loid)s.

## Methodology

### Study area and population

The study was carried out in Santander Bay, Cantabria, Northern Spain (about 250,000 inhabitants in 2019). Among the local industrial sources of metal(loid)s in this area, a ferromanganese alloy plant is the main emitter of metal(loid)s, mainly of Mn as reported by Otero-Pregigueiro et al. (2018), outstanding relatively high levels of Mn in ambient air that frequently exceeded the WHO guideline value (150 ng/m^3^, annual mean) in the town of Maliaño (about 10,000 inhabitants), where the ferromanganese alloys smelter is located (Hernández-Pellón & Fernández-Olmo, 2019).

**Fig. 1:**
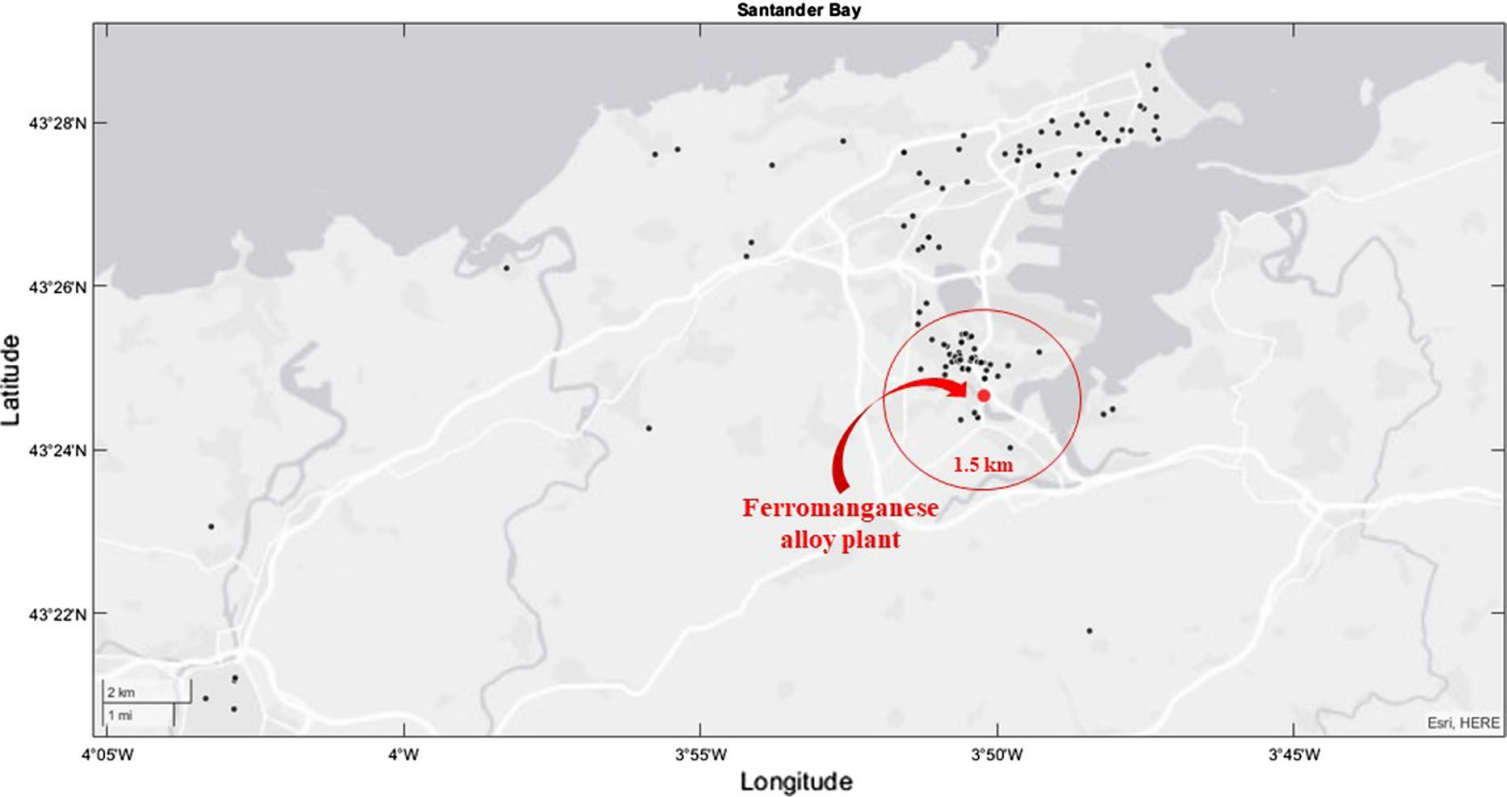
Location of participants’ residence and the Mn alloy plant

Volunteers were recruited as specified in Ruiz-Azcona et al. (2021). All volunteers considered in this study were over 18 years old, and without previous or current work in relation with the ferromanganese plant or any other occupational exposure to Mn. All of them resided for a minimum of one year in Santander Bay at different distances from the ferroalloy factory, which was intentionally placed in Fig. 1 in the centre of the exposure area, to account for the degree of exposure to Mn and the other studied metal(loid)s. The study population was then divided into highly exposed (distance less than 1.5 km between each volunteer’s residence and the Mn alloy plant, i.e. those living in Maliaño, where the main Mn source is located) and moderately exposed (distance greater than 1.5 km, i.e. those living outside Maliaño, mainly in the city of Santander).

The study was approved by the ethical committee of clinical research in Cantabria (CEIC) and by the ethical committee of research of the University of Cantabria (CEUC).

Written informed consent was obtained from each subject and whole blood, scalp hair and fingernails collection was performed only after permission was obtained. In addition to biological samples, PM samples were obtained from personal samplers that were worn for 24 h by each volunteer. Subjects were asked to complete a structured questionnaire containing socio-demographic characteristics, health and medication status, occupational data, smoking habits, dietary habits including daily intake of Mn-rich foods and Mn food supplements, alcohol consumption and other lifestyles. The sampling campaign started in November 2019 and ended in November 2020 (interrupted from March 2020 to June 2020 due to the COVID-19 outbreak), concluding the sampling campaign with a total of 130 volunteers.

### Sample preparation

All the used reagents for treatment and sample preparation were of analytical grade provided by Merck and PanReac AppliChem (Darmstadt, Germany). Whole blood samples of approximately 7.5 mL were collected by venipuncture after disinfection of the skin with 70% alcohol, collected in lithium heparin monovettes developed for metal determination (Sarstedt, Nümbrecht, Germany). These samples were refrigerated for up to 14 days until dilution with an alkaline solution (2% (w/v) 1-butanol, 0.05% (w/v) EDTA, 0.05% (w/v) triton X-100 and 1% (w/v) NH_4_OH) as described in González-Antuña et al. (2017) at a minimum whole blood/alkaline solution ratio of 1/10 (w/w), and subsequent analysis by ICP/MS. Scalp hair and fingernail samples were collected in polypropylene flasks using clean ceramic scissors and nail clippers, respectively. A tuft of hair was cut from the occipital part of the head, using the 2 cm closest to the scalp for analysis. By using only 2 cm from the root of the hair, alterations in hair morphology due to dyes are eliminated. This is because the hair tips may contain higher levels of metals since the hair further from the root has longer contact with the environment and additional dye applications. Each additional dye application process leads to a higher level of oxidation of the hair, thus creating more potential binding sites, and together with the additional mechanical wear that occurs when moving from root to tip, can facilitate the diffusion of dyes into the hair (Godfrey et al., 2013).

Fingernails of both hands were cut after asking the volunteers to wash them with the liquid soap provided and to rinse them thoroughly with tap water. Females were asked whether they dyed their hair and/or polished their fingernails. When they polished them, the polish residues were removed using acetone before cutting, and the fingernails were sonicated for 10 min before proceeding to the washing protocol. Scalp hair and fingernails were washed according to the washing protocol described in Eastman et al. (2013), thus removing all exogenous metals and ensuring that only endogenous metals were quantified. Once cleaned, samples were microwave digested (Milestone, Ethos One) at 200 °C in a 4/1 (v/v) HNO_3_/H_2_O_2_ solution, and finally analysed by ICP/MS.

A personal two-stage modular impactor (SKC PMI coarse) was used to collect PM_2.5_ and PM_10-2.5_ samples for 24 h, connected to a personal pump (SKC Aircheck XR5000) operating at a flow rate of 3 lpm. PTFE filters of 37 mm (1 µm pore size) were used for the PM_2.5_ fraction, and PTFE filters of 25 mm (1 µm pore size) were used for the PM_10-2.5_ fraction (SKC Inc., Houston, USA). An in vitro bioaccessibility test was performed by extracting each filter with 10 mL of ALF (Artificial Lysosomal Fluid) as lung fluid for 24 h for PM_2.5_ filters, and gastric fluid for 1 h for PM_10-2.5_ filters, in an incubation system (MRHX-04, LSCI) at 37 °C with end-to-end rotation (SBS) at 30 rpm. After the leaching assay, the samples were centrifuged (Mistasel-BL/Selecta) and the supernatants were filtered using polypropylene syringe filters with a pore size of 0.45 µm. The choice of fluids and composition is described in detail in Expósito et al. (2021). Then, the insoluble fraction (non-bioaccessible fraction) was digested based on the European standard method "EN-UNE 14,902:2006", which consisted of an acid digestion of each filter in a HNO_3_/H_2_O_2_ solution in a 4/1 (v/v) ratio, up to 220 °C. The ALF extracts were stored until analysis at 4 °C for a maximum of 48 h.

### Metal(loid) analysis

Mass spectrometry inductively coupled plasma (ICP/MS, Agilent 7500 CE) was used for the analysis of ^55^Mn, ^56^Fe, ^63^Cu, ^66^Zn, ^75^As, ^111^Cd and ^207^Pb in the different samples. Internal standards (^89^Y, ^103^Rh and ^185^Re) were added to each vial to correct for instrumental drifts, and a collision cell with a helium flow rate of 4.8 mL/min was used to minimise spectral interferences. Since whole blood, ALF and gastric fluid can cause spectral and non-spectral interferences (matrix effects) during ICP/MS analysis, the determination of the concentration of the metals studied in these samples was performed by adding the same solution to the Multi-Element Standard Solution used to calibrate the instrument. In the case of samples from acid digestion, the calibration standards were simply prepared in dilute nitric acid (1 N). Seven calibration points between 0 and 25 ppb were used and samples were diluted when necessary.

Limits of detection (LOD) were calculated based on the variability of 10 procedural blanks (two-tailed Student's *t*-test with 95% confidence for n-1 samples (2.26) times the standard deviation of the blanks), being constant for each metal(loid) for whole blood and filters, and variable for scalp hair and fingernails, depending on the weight of sample from each volunteer. Limit of quantification was determined as 10 times the SD of blanks (Al-Hakkani, 2019) (see Supplementary Table 1). Measurements of these procedural blanks also allowed checking for possible contamination of containers and reagents. The mean values of the studied metal(loid)s measured in these blanks were subtracted from all samples except for whole blood because both calibration points and samples were prepared in the same solution. Results were presented for elements with a minimum of 50% of the samples above the limit of detection.

The analytical methods described were validated using certified reference material for whole blood and human hair (Seronorm™ Trace Elements Whole Blood L-1 and ERM®-DB001, respectively), but no reference material was found for fingernails. The recoveries obtained for the different certified metal(loid)s range from 94 to 110% for whole blood and from 93 to 110% for hair (see Supplementary Table 2). A worse recovery was obtained for Mn in hair (144%), but its metal content was not certified in ERM®-DB001, because only one laboratory provided results on hair Mn concentration. The digestion method was validated in the research group as described in Hernández-Pellón et al. (2018). In addition to the use of certified reference materials, after calibration and at the end of each analytical run, quality control standards covering the concentration range of interest were measured to check the accuracy of the measurements.

### Wind-weighted distance calculation procedure for exposure metrics

To study the correlation between the levels of metal(loid)s in the short-term markers (whole blood and PM) and the distance between each volunteer’s residence and the main metal(loid) source weighted by wind, hourly wind data were taken for the personal sampling time slots of each subject. For this purpose, hourly wind direction and speed data from the Santander—Parayas Airport weather station provided by Openair thorough the World Met package (R version 4.0.5) were used.

The procedure described by Haynes et al. (2012) was followed to calculate the wind-weighted source/home distance. First, Wind Index (WI), a parameter between 0–1 indicating whether the subject is upwind (0) or downwind (1) of the source, was calculated, according to Eq. (1), where *α*_homesource_ is the direction of the home from the source (rad) and *θ*_m_ represents the average wind direction at the selected meteorological station in the sampling period in which the volunteer wore the personal sampler (rad):

1 $${\text{WI}} = \frac{{1 - \cos \left( {\alpha_{{{\text{homesource}}}} - \theta_{{\text{m}}} } \right)}}{2}$$

The average wind direction (*θ*_m_) was calculated from hourly data considering the wind speed. To calculate the average direction in each period, it was necessary to first determine the *U*_mean_ and *V*_mean_ components from Eqs. 2 and 3, where *N* is number of hours of sampling, *u*_*i*_ is the hourly wind speed (m/s) and *θ*_*i*_ is the hourly wind direction (rad).

2 $$U_{{{\text{mean}}}} = - \frac{1}{N}\sum u_{i} \;sen\left( {\theta_{i} } \right)$$

3 $$V_{{{\text{mean}}}} = - \frac{1}{N}\sum u_{i} \cos \left( {\theta_{i} } \right)$$

To obtain the average direction (*θ*_m_), Eq. 4 was applied (Grange, 2014), where FLOW =  + 180 when ArcTan < 180 and FLOW =  − 180 when ArcTan > 180:

4 $$\theta_{{\text{m}}} = {\text{ArcTan}}\left( {\frac{{U_{{{\text{mean}}}} }}{{V_{{{\text{mean}}}} }}} \right) + {\text{FLOW}}$$

Then, the weighted distance (*d*_w_) was calculated for each subject by dividing the actual distance (*d*) by the WI, as shown in Eq. (5).

5 $$d_{{\text{w}}} = \frac{d}{{{\text{WI}}}}$$

With respect to the long-term markers, to calculate the weighted source/home distance, wind data were taken for the months prior to fingernails and scalp hair sampling for each subject (6 months for fingernail samples and 2 months for scalp hair samples). For these longer periods, the procedure shown in Eqs. (1–5) sometimes failed, due to the lack of representativeness of the average wind direction over these periods. To address this, an alternative procedure was developed to calculate the weighted distance (*d*′_w_), using a weighting factor (*w*_f_) that considers the frequency with which the wind blows downwind (i.e. from the source to the residence) during the period considered for each subject. For this purpose, the area of interest was divided into 8 sectors, placing the emitting source in the centre of the area and assigning each residence a wind sector. Next, the frequency (fraction of hours) in which the wind blew from the sector opposite the volunteer’s home was calculated with respect to the total number of hours. Finally, the distance between the source and the subject's home was weighted by dividing the raw distance by this weighting factor (Eq. 6).

6 $$d_{{\text{w}}}^{\prime } = \frac{d}{{w_{{\text{f}}} }}$$

### Statistical analysis

Data analysis was performed with IBM SPSS Statistics software (version 22) and R software (version 4.0.5). Statistical analysis was only performed when at least 50% of the data above the LOD was available. For metal(loid) concentrations in biomarkers and PM samples below the LOD, a value of LOD/2 was assigned.

All quantitative variables in the study were tested for normality using the Kolmogorov–Smirnov test with Lilliefors correction, and the homogeneity of variances was tested using Levene's test. Student's *t*-test (normal distribution and homogeneity of variances), Welch's *t*′-test (normal distribution and non-homogeneous variances) and the Mann–Whitney *U* test (non-normal distribution) were used to compare means/medians between the established groups.

To study the potential effect of confounders on the differences between groups as a function of source distance (i.e. highly exposed vs. moderately exposed), linear regression models were used to calculate crude and adjusted Mean Differences (MDs) of each metal(loid) concentration with their 95% confidence intervals (CI). Age (as a continuous variable), sex and study level (ordinal categorised) were pre-established as confounders and included in a first multivariate model. Also, a second multivariate model added as confounders employment status, tobacco smoking and dietary habits: Mn supplement intake and high Mn food consumption (nuts, tea ≥ 5/week, fish as tunas or salmon families… ≥ 3/week).

Spearman correlation coefficients were calculated between the metal(loid)s of each matrix and between the matrices for each metal(loid). Finally, correlations were also calculated between the biomarkers studied, the levels in PM and the wind-weighted distance between each volunteer’s residence and the main metal(loid) source, as well as the age of the subjects. The Chi-square test was applied to determine whether there was any relationship between exposure (categorised as highly/moderately exposed) and the categorical variables used in the description of the population (sex, education level, smoking status). All tests were bilateral, and the alpha error was set at 5%.

## Results

### Description of the study population

Table 1 shows the socio-demographic characteristics of the studied population divided according to exposure (moderately exposed/highly exposed) with an age range between 20 and 71 years, with an average of 41.75 ± SD = 13.97 years. The highly exposed population lives within a radius of 0.8 km (0.25–1.5 km) from the ferromanganese factory as the main source of contamination, while the moderately exposed population resides within an average radius of 7.3 km (2–34 km). Significant differences were seen between exposure by years of residence, higher for the most exposed group (*p* < 0.001); by educational level, lower for the most exposed population (*p* = 0.005); and by employment status, with more people employed full time in the moderately exposed population (*p* = 0.045). However, no differences were seen by sex and smoking habits or alcohol consumption. Regarding to diet, none participant was vegetarian and the consumption of grains, greenpeas and beans was similar. Furthermore, the study population comes from an urban/industrial area, so their diet is mainly based on products purchased in supermarkets, thus ruling out the intake of potentially contaminated food grown in local soils. In addition, the ingestion of water was not considered because of the low metal(loid) content determined in local tap water. The rest of dietary characteristics are also included in Table 1.

**Table 1:** Socio-demographic characteristics of the study population divided according to the exposure groups (highly exposed (HE) (≤ 1.5 km) versus moderately exposed (ME) (> 1.5 km)

Characteristics	ME (> 1.5 km) *N* = 65		HE (≤ 1.5 km) *N* = 65		Total *N* = 130		*p*-value
Source distance from main point (m)							
Arithmetic Mean, SD	7294.68	5258.64	799.24	297.94	4046.96	4938.92	
Geometric Mean	6212.08		747.87		2155.42		
Median, P95	6085.55	18782.14	743.19	1447.45	1770.40	17910.06	
Range: min, max	2040.80	33984.74	268.07	1500.00	268.07	33984.74	
Interquartile Range (P25, P75)	4969.84	7254.11	594.43	946.25	728.56	6089.44	
Age							
Arithmetic Mean, SD	39.77	13.45	43.66	14.31	41.72	13.97	0.092**
Range: min, max	20	71	20	71	20	71	
Years residing							
Arithmetic Mean, SD	11.62	12.42	18.85	13.96	15.23	13.65	< 0.001**
Range: min, max	1	60	1	71	1	71	
Sex (*n*, %)							0.553*
Female	46	70.8%	49	75.4%	95	100.0%	
Male	19	29.2%	16	24.6%	35	100.0%	
Studies (*n*, %)							0.005*
Primary education	3	4.6%	3	4.6%	6	4.6%	
Secondary Education/Vocational education and Training	7	10.8%	15	23.1%	22	16.9%	
High school level/Certificate of Higher Education	10	15.4%	20	30.8%	30	23.1%	
University studies (Bachelor’s Degree)	8	12.3%	11	16.9%	19	14.6%	
University studies (University Degree)	37	56.9%	16	24.6%	53	40.8%	
Employment status (*n*, %)							0.045*
Employed full time	54	83.1%	43	66.2%	97	74.6%	
Unemployed	1	1.5%	4	6.2%	5	3.8%	
Housewife	0		5	7.7%	5	3.8%	
Retired	7	10.8%	6	9.2%	13	10.0%	
Full-time student	3	4.6%	7	10.8%	10	7.7%	
Smoking status (*n*, %)							0.498*
Non-smoker	42	64.6%	42	64.6%	84	64.6%	
Former	9	13.8%	13	20.0%	22	16.9%	
Current	14	21.5%	10	15.4%	24	18.5%	
Alcohol status							0.597*
Never	34	52.3%	37	56.9%	71	54.6%	
Ever	31	47.7%	28	43.1%	59	45.4%	
Average of pure ethanol (g/week) (*n*, %)							0.245*
0 g/week	34	52.3%	37	56.9%	71	54.6%	
1–24 g/week	11	16.9%	11	16.9%	22	16.9%	
25–74 g/week	16	24.6%	17	26.2%	33	25.4%	
≥ 75 g/week	4	6.2%	0	0.0%	4	3.1%	
Mn food supplements intake (*n*, %)							0.154*
No	63	96.9%	65	100.0%	128	98.5%	
Yes	2	3.1%	0	0.0%	2	1.5%	
Nuts ≥ 5/week (*n*, %)							0.456*
No	57	87.7%	54	83.1%	111	85.4%	
Yes	8	12.3%	11	16.9%	19	14.6%	
Tea ≥ 5/week (*n*, %)							0.019*
No	59	90.8%	49	75.4%	108	83.1%	
Yes	6	9.2%	16	24.6%	22	16.9%	
Fish as tunas or salmon ≥ 3/week (*n*, %)							0.784*
No	58	89.2%	57	87.7%	115	88.5%	
Yes	7	10.8%	8	12.3%	15	11.5%	

Bold implies that the *p*-value is significant

^*^Chi-square test

^**^Mann–Whitney *U* test

### (Bio)markers

Tables 2 and 3 show the concentration of the studied metal(loid)s by sex (arithmetic mean (AM), standard deviation (SD), median and reference values (RV) with 95% confidence interval) in the short-term (bio)markers (personal filters and whole blood), as well as in the long-term biomarkers (scalp hair and fingernails), respectively. Metal(loid)s with at least 50% values above the LOD in each matrix are shown. Reference values were calculated for biological matrices only, as the 95^th^ percentile with a 95% confidence interval, as specified by Saravanabhavan et al. (2017). With respect to whole blood as short-term biomarker, significant sex differences were observed for Fe (*p* = 0.039), Cu (*p* = 0.006) and Pb (*p* = 0.001), with higher concentrations of Fe and Pb for males (492,650 µg/L vs. 466,302 µg/L and 11.40 µg/L vs. 8.49 µg/L, respectively), and higher levels of Cu in females (824.5 µg/L vs. 716.4 µg/L). Regarding to long-term biomarkers, significant sex differences were shown for Mn (*p* = 0.008), Cu (*p* = 0.021), Zn (*p* = 0.009), Cd (*p* = 0.020) and Pb (*p* = 0.007) in scalp hair, with higher concentrations for all metals except Cu in males (10,639 ng/g vs. 8450 ng/g), while no significant differences for any metal in the case of fingernails were found. The possible influence of nail varnish on the metal levels was not considered, since only four female volunteers had polished fingernails. In addition, according to the research of Ceballos et al. (2021), the internal levels of metal(loid)s measured in nail technicians’ toenails were comparable to those reported in other studies in females, except for antimony, arsenic, chromium, mercury, and nickel.

**Table 2:** Summary statistics for short-term (bio)markers (filters and whole blood) by sex

(Bio)marker	Female				Male				*p*-value	Total			
	*N*	AM (SD)	Median	RV (95% CI)	*N*	AM (SD)	Median	RV (95% CI)		*N*	AM (SD)	Median	RV (95% CI)
Mn coarse fraction bioaccessible (ng/m^3^)	95	68.18 (204.49)	15.17		35	41.92 (72.82)	10.57		0.527	130	61.11 (178.90)	13.61	
Mn coarse fraction non-bioaccessible (ng/m^3^)	95	12.73 (43.07)	3.40		35	9.36 (12.16)	2.62		0.862	130	11.82 (37.33)	3.39	
Mn coarse fraction total (ng/m^3^)	95	80.91 (246.81)	16.51		35	51.28 (82.10)	15.46		0.723	130	79.93 (215.26)	16.47	
Mn fine fraction bioaccessible (ng/m^3^)	95	70.07 (147.06)	17.82		35	53.12 (100.09)	12.85		0.680	130	66.31 (135.79)	17.05	
Mn fine fraction non-bioaccessible (ng/m^3^)	95	14.02 (23.27)	5.73		35	8.91 (9.98)	6.61		0.858	130	12.64 (20.64)	5.80	
Mn fine fraction total (ng/m^3^)	95	84.08 (159.31)	25.26		35	65.03 (102.57)	20.02		0.937	130	78.95 (146.07)	25.00	
Mn total fraction (PM10) (ng/m^3^)	95	165.00 (376.19)	42.13		35	116.31 (155.90)	51.53		0.927	130	151.89 (331.66)	43.87	
Fe coarse fraction bioaccessible (ng/m^3^)	95	62.66 (101.29)	36.30		35	32.96 (28.96)	26.10		0.112	130	54.66 (88.72)	31.65	
Fe fine fraction non-bioaccessible (ng/m^3^)	95	118.42 (244.32)	65.40		35	79.03 (82.99)	62.00		0.869	130	107.81 (213.59)	65.05	
Pb fine fraction bioaccessible (ng/m^3^)	95	13.24 (21.10)	4.50		35	11.86 (13.71)	7.40		0.634	130	12.87 (19.34)	5.25	
Blood Mn (µg/L)	95	10.04 (3.14)	9.76	15.89 (15.26–16.53)	35	9.71 (4.01)	9.01	18.51 (17.18–19.84)	0.375	130	9.95 (3.38)	9.58	16.01 (15.43–16.59)
Blood Fe (µg/L)	95	485327 (81231)	466302	668017 (651682–684351)	35	505143 (56523)	492650	633413 (614687–652139)	**0.039**	130	490662 (75684)	478263	654062 (641052–667072)
Blood Cu (µg/L)	95	886.9 (248.1)	824.5	1425.7 (1375.8–1475.6)	35	770.2 (167.7)	716.4	1142.9 (1087.4–1198.5)	**0.006**	130	855.5 (234.4)	799.8	1368.3 (1328.0–1408.6)
Blood Zn (µg/L)	95	6071 (1897)	5799	9538 (9157–9920)	35	6150 (1705)	5596	10187 (9622–10752)	0.923	130	6093 (1841)	5746	9964 (9347–9980)
Blood As (µg/L)	95	4.68 (5.36)	3.06	18.38 (17.30–19.45)	35	5.02 (5.26)	3.04	21.63 (19.89–23.37)	0.797	130	4.77 (5.31)	3.06	18.90 (17.98–19.81)
Blood Pb (µg/L)	95	9.74 (5.52)	8.49	19.52 (18.41–20.63)	35	14.53 (9.17)	11.40	40.24 (37.20–43.27)	**0.001**	130	11.03 (6.99)	9.13	24.82 (23.62–26.02)

Bold implies that the *p*-value is significant

**Table 3:** Summary statistics for long-term biomarkers (scalp hair and fingernails) by sex

Biomarker	Female				Male				*p*-value	Total			
	*N*	AM (SD)	Median	RV (95% CI)	*N*	AM (SD)	Median	RV (95% CI)		*N*	AM (SD)	Median	RV (95% CI)
Scalp hair Mn (ng/g)	93	220.5 (205.4)	168.3	721.7 (680.0–763.5)	29	366.3 (427.2)	295.9	1550.5 (1395.1–1706.0)	**0.008**	122	255.2 (279.6)	185.1	719.0 (669.3–768.6)
Scalp hair Fe (ng/g)	93	9997 (15188)	6728	30010 (26923–33096)	29	26519 (58257)	8615	233860 (212657–255063)	0.094	122	13925 (31790)	6823	45887 (40246–51528)
Scalp hair Cu (ng/g)	93	14175 (14387)	10639	40706 (37782–43630)	29	9658 (5850)	8450	26932 (24262–28521)	**0.021**	122	13101 (13001)	9787	35516 (33209–37823)
Scalp hair Zn (ng/g)	93	123641 (47488)	124375	199111 (189460–208763)	29	164080 (89082)	150307	434800 (402378–467222)	**0.009**	122	133253 (62046)	130098	218907 (207897–229917)
Scalp hair Cd (ng/g)	93	12.52 (23.47)	6.74	37.21 (32.44–41.98)	29	16.31 (15.31)	10.98	54.75 (49.18–60.32)	**0.020**	122	13.42 (21.81)	7.57	48.09 (44.22–51.96)
Scalp hair Pb (ng/g)	93	210.9 (214.6)	139.0	669.5 (625.9–713.1)	29	361.5 (368.7)	234.0	1387.2 (1253.0–1521.4)	**0.007**	122	246.7 (265.7)	148.2	861.1 (813.9–908.2)
Fingernails Mn (ng/g)	87	967.5 (1097.1)	562	3778 (3547–4008)	29	844.3 (1203.3)	532	4331 (3893–4769)	0.337	116	936.7 (1120.5)	555	3549 (3345–3753)
Fingernails Fe (ng/g)	87	28365 (31490)	19187	93071 (86454–99687)	29	28776 (29301)	18363	101105 (90441–111769)	0.690	116	28468 (30832)	18746	90461 (84850–96072)
Fingernails Cu (ng/g)	73	5671 (9904)	3544	15626 (13355–17898)	23	4730 (2887)	3840	13412 (12232–14592)	0.328	96	5446 (8743)	3634	14470 (12721–16219)
Fingernails Zn (ng/g)	87	135378 (55913)	124375	227144 (215395–238893)	29	117672 (34975)	150307	207368 (194639–220097)	0.064	116	130952 (51913)	118442	220002 (210555–229449)
Fingernails Pb (ng/g)	73	127.5 (208.5)	97.3	402.0 (354.2–449.9)	23	128.5 (119.3)	94.3	474.1 (425.3–522.8)	0.866	96	150.5 (190.8)	96.0	424.8 (386.6–462.9)

Bold implies that the *p*-value is significant

**Fig. 2:**
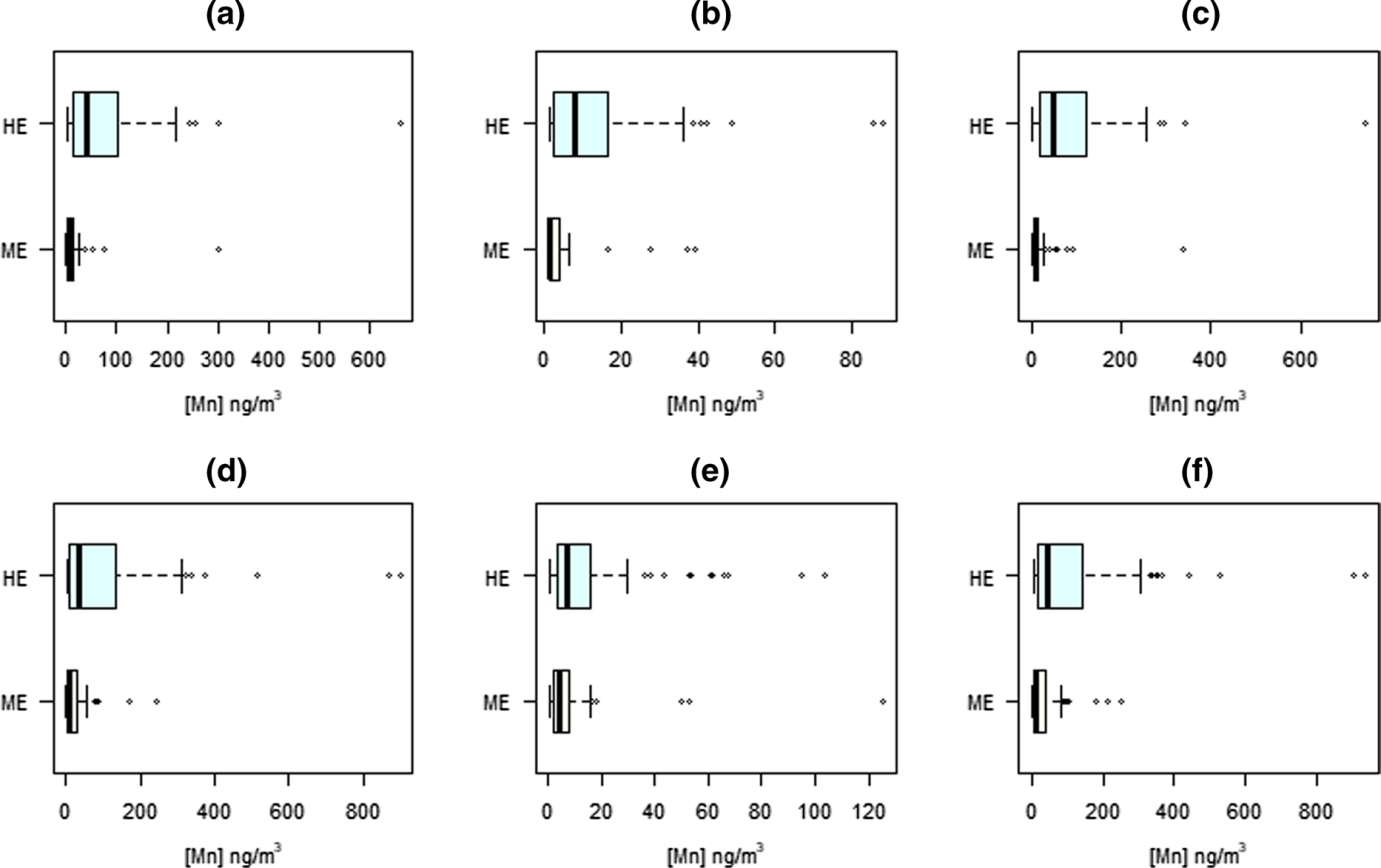
Levels of Mn in filters from personal sampling according to the exposure to the main Mn source: moderately exposed (ME) versus highly exposed (HE): (**a**) bioaccessible, PM_10-2.5_; (**b**) non-bioaccessible, PM_10-2.5_; (**c**) total, PM_10-2.5_; (**d**) bioaccessible, PM_2.5_; (**e**) non-bioaccessible, PM_2.5_; (**f**) total, PM_2.5_

**Fig. 3:**
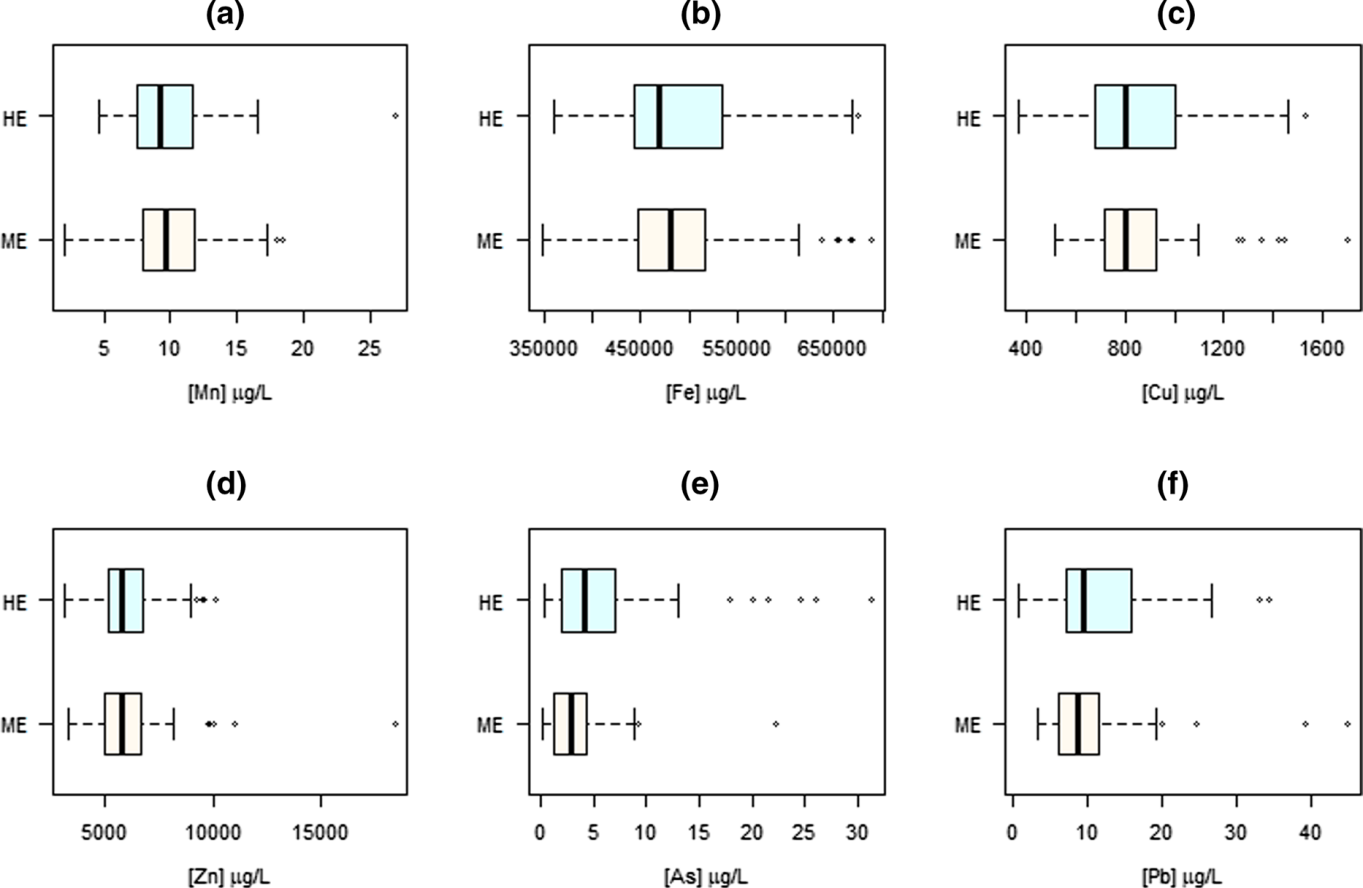
Levels of metal(loid)s in whole blood as short-term biomarker according to the exposure to the main Mn source: moderately exposed (ME) versus highly exposed (HE): (**a**) Mn; (**b**) Fe; (**c**) Cu; (**d**) Zn; (**e**) As; (**f**) Pb

**Fig. 4:**
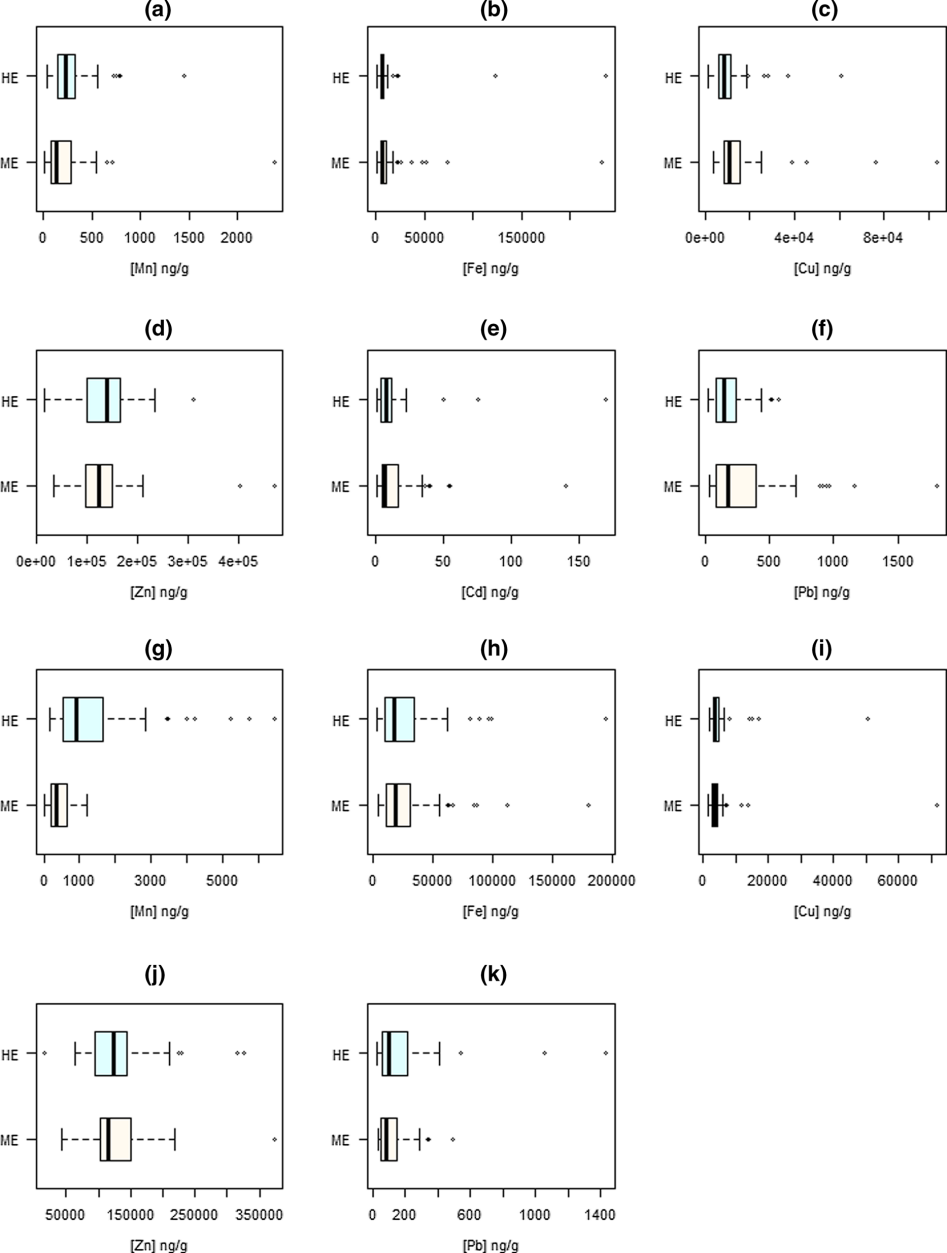
Levels of metals in long-term biomarkers according to the degree of exposure to the main Mn source: moderately exposed (ME) versus highly exposed (HE): (**a**) scalp hair Mn; (**b**) scalp hair Fe; (**c**) scalp hair Cu; (**d**) scalp hair Zn; (**e**) scalp hair Cd; (**f**) scalp hair Pb; (**g**) fingernails Mn; (**h**) fingernails Fe; (**i**) fingernails Cu; (**j**) fingernails Zn; **k**) fingernails Pb

Figures 2, 3 and 4 show the levels of the selected metal(loid)s in the studied short- and long-term indicators, according to the degree of exposure (highly vs. moderately exposed). In terms of exposure, significant differences of medians were only observed for As in whole blood (*p* = 0.002), Mn and Cu in scalp hair (*p* = 0.008 and 0.001, respectively) and Mn and Pb in fingernails (*p* < 0.001 and 0.025, respectively), all concentrations being higher for the highly exposed group with the exception of Cu in scalp hair. For filters, the most exposed population showed significantly higher concentrations for all the Mn fractions analysed (PM_10-2.5_ and PM_2.5_, both bioaccessible and non-bioaccessible), as observed in Fig. 2. These differences were maintained statistically significant after adjusting for the potential confounders mentioned in Methodology, as shown in Table 4, except for Cu in scalp hair and Mn in the non-bioaccessible fine fraction, which lost significance in the multivariate models.

**Table 4:** Crude and adjusted Mean Differences (MD)s for metal(loid)s levels between highly and moderately exposed groups

Short-term exposure	Highly versus moderately exposed											
	MDcrude	95% CI		*p* value	MDa_1_	95% CI		*p* value	MDa_2_	95% CI		*p* value
*PM personal samplers* (ng/m^3^)												
Coarse fraction (PM_10-2.5_) Mn												
Bioaccessible	93.00	32.83	153.18	**0.003**	82.42	18.99	145.85	**0.011**	89.88	22.84	156.92	**0.009**
Non-Bioaccessible	15.60	2.88	28.31	**0.017**	14.67	1.21	28.14	**0.033**	16.71	2.49	30.93	**0.022**
Total	108.60	36.04	181.16	**0.004**	97.09	20.53	173.65	**0.013**	106.59	25.67	187.51	**0.010**
Fine fraction (PM_2.5_) Mn												
Bioaccessible	85.78	40.91	130.65	** < 0.001**	88.22	41.41	135.03	** < 0.001**	90.03	41.92	138.13	** < 0.001**
Non-Bioaccessible	8.64	1.61	15.67	**0.016**	6.30	− 0.95	13.54	0.088	4.38	− 2.57	11.34	0.214
Total	94.42	46.28	142.56	** < 0.001**	94.52	44.34	144.69	** < 0.001**	106.59	25.67	187.51	**0.010**
*Total (PM*_*10*_*) Mn*	203.02	93.05	312.99	** < 0.001**	191.61	75.67	307.54	**0.001**	201.00	78.58	323.42	**0.001**
Coarse fraction (PM_10-2.5_) Fe												
Bioaccessible	35.94	5.65	66.23	**0.020**	28.94	− 2.63	60.50	0.072	30.63	− 2.27	63.53	0.068
Fine fraction (PM_2.5_) Fe												
Non-Bioaccessible	24.15	− 50.15	98.45	0.521	18.38	− 60.02	96.78	0.643	− 8.30	− 89.28	72.68	0.840
Fine fraction (PM_2.5_) Pb												
Bioaccessible	5.19	− 1.49	11.87	0.127	3.75	− 3.27	10.77	0.293	3.68	− 3.56	10.91	0.317
Blood (µg/L)												
Blood Mn	0.18	− 1.00	1.36	0.759	0.52	− 0.71	1.75	0.403	0.42	− 0.85	1.70	0.513
Blood Fe	− 7159.30	− 33500.51	19181.92	0.592	− 1212.46	− 28783.72	26358.79	0.931	7210.15	− 21596.27	36016.57	0.621
Blood Cu	− 7.86	− 89.54	73.82	0.849	6.10	− 76.71	88.91	0.884	− 13.78	− 95.38	67.82	0.739
Blood Zn	− 67.65	− 709.11	573.81	0.835	− 74.33	− 750.22	601.56	0.828	24.75	− 676.75	726.25	0.944
Blood As	2.79	1.01	4.58	**0.002**	2.24	0.48	4.01	**0.013**	2.05	0.22	3.88	**0.028**
Blood Pb	1.24	− 1.19	3.67	0.314	0.63	− 1.58	2.84	0.573	0.75	− 1.45	2.94	0.502
*Long-term exposure*												
Scalp hair (ng/g)												
Scalp hair Mn	299.76	− 207.48	806.99	0.244	311.02	− 209.23	831.27	0.239	371.24	− 177.56	920.03	0.183
Scalp hair Fe	− 1574.27	− 12962.60	9814.06	0.785	1372.38	− 10245.03	12989.80	0.815	1520.24	− 10804.21	13844.70	0.807
Scalp hair Cu	− 4732.05	− 9296.41	− 167.70	**0.042**	− 4034.22	− 8700.45	632.02	0.090	− 3422.19	− 8374.47	1530.09	0.174
Scalp hair Zn	7099.71	− 15084.49	29283.91	0.528	12442.24	− 9882.76	34767.23	0.272	15850.70	− 3814.92	35516.33	0.113
Scalp hair Cd	− 1.70	− 9.48	6.08	0.666	− 1.74	− 9.91	6.43	0.674	− 0.66	− 9.27	7.94	0.879
Scalp hair Pb	− 64.09	− 225.73	97.55	0.434	− 44.34	− 208.08	119.39	0.593	− 25.24	− 196.43	145.95	0.771
Fingernails (ng/g)												
Fingernails Mn	1006.68	637.21	1376.14	** < 0.001**	839.68	459.68	1219.68	** < 0.001**	861.31	467.36	1255.26	** < 0.001**
Fingernails Fe	− 222.52	− 11613.92	11168.88	0.969	− 1030.66	− 13153.73	11092.42	0.867	774.68	− 11971.86	13521.21	0.904
Fingernails Cu	333.81	− 3240.20	3907.83	0.853	1065.19	− 2763.15	4893.53	0.582	1440.86	− 2729.58	5611.31	0.494
Fingernails Zn	878.48	− 18301.33	20058.29	0.928	− 1950.18	− 22105.12	18204.75	0.848	− 3036.20	− 24646.57	18574.16	0.781
Fingernails Pb	74.32	− 2.20	150.84	0.057	75.86	− 6.80	158.51	0.072	102.06	14.21	189.91	**0.023**

Bold implies that the *p*-value is significant

MD = Mean Difference. MD_a1_ = MD adjusted for age, sex, and study level. MD_a2_ = MD adjusted for age, sex, study level, employment status, tobacco smoking and dietary habits (Mn supplement intake and food with Mn consumption (Nuts, Tea ≥ 5/week, Fish as tunas, salmon families… ≥ 3/week). A positive MD indicates higher levels on participants living in a shorter distance from the ferroalloy plant

### Correlation analysis

Spearman’s correlation coefficients between the concentration of metal(loid)s in each matrix (filters, whole blood, scalp hair and fingernails) and wind-weighted distance are shown in Table 5.

**Table 5:** Spearman’s correlation coefficients between concentrations of all employed (bio)markers and wind-weighted distance; markers are shown in two groups according to the distance weighting methods used: short-term and long-term

	*r*	*p*-value
*Short-term (bio)markers*		
Mn coarse fraction bioaccessible (ng/m^3^)	− 0.609	** < 0.001**
Mn coarse fraction non-bioaccessible (ng/m^3^)	− 0.44	** < 0.001**
Mn coarse fraction total (ng/m^3^)	− 0.612	** < 0.001**
Mn fine fraction bioaccessible (ng/m^3^)	− 0.449	** < 0.001**
Mn fine fraction non-bioaccessible (ng/m^3^)	− 0.319	**0.001**
Mn fine fraction total (ng/m^3^)	− 0.439	** < 0.001**
Mn total (PM_10_) (ng/m^3^)	− 0.549	** < 0.001**
Fe coarse fraction bioaccessible (ng/m^3^)	− 0.343	** < 0.001**
Fe fine fraction non-bioaccessible (ng/m^3^)	− 0.150	0.114
Pb fine fraction bioaccessible (ng/m^3^)	− 0.179	0.057
Whole blood Mn (µg/L)	0.055	0.563
Whole blood Fe (µg/L)	0.065	0.495
Whole blood Cu (µg/L)	0.152	0.109
Whole blood Zn (µg/L)	0.028	0.766
Whole blood As (µg/L)	− 0.239	**0.011**
Whole blood Pb (µg/L)	− 0.063	0.506
*Long-term biomarkers*		
Scalp hair Mn (ng/g)	− 0.104	0.291
Scalp hair Fe (ng/g)	0.269	**0.006**
Scalp hair Cu (ng/g)	0.311	**0.001**
Scalp hair Zn (ng/g)	− 0.207	**0.035**
Scalp hair Cd (ng/g)	0.151	0.127
Scalp hair Pb (ng/g)	0.300	**0.002**
Fingernails Mn (ng/g)	− 0.607	** < 0.001**
Fingernails Fe (ng/g)	0.041	0.682
Fingernails Cu (ng/g)	− 0.039	0.725
Fingernails Zn (ng/g)	− 0.032	0.754
Fingernails Pb (ng/g)	− 0.344	** < 0.001**

Bold implies that the *p*-value is significant

For short-term markers, PM-bound Mn showed a significant negative correlation for all the analysed fractions with respect to the weighted distance from the most important source, in which those subjects who live closest to the Mn alloy factory (i.e. in the municipality of Maliaño) were more exposed. Table 5 also shows the lack of correlation between Mn in whole blood and weighted distance (*r* = 0.055, *p* = 0.563). On the other hand, a significant negative correlation of As in whole blood was found with distance (*r* =  − 0.239, *p* = 0.011), but as we will see later, it is not possible to confirm that inhalation was the main route of exposure, since its levels in PM filters were below the LOD.

For long-term biomarkers, Mn showed negative correlations with weighted distance, in agreement with that shown in Fig. 4 for both scalp hair and fingernails, respectively. However, this correlation was only statistically significant for fingernails (*r* =  − 0.607, *p* < 0.001). A significant negative correlation with distance to the source was also observed for Zn in scalp hair (*r* =  − 0.207, *p* = 0.035), but not in fingernails. Copper and Fe show no correlation with distance in fingernails, but a positive and significant correlation in scalp hair: Fe (*r* = 0.269, *p* = 0.006), Cu (*r* = 0.311, *p* = 0.001). With respect to Pb, contradictory results were found: a positive correlation with distance in scalp hair and negative in fingernails, both significant. Negative but no significant correlations were obtained in the bioaccessible fine fraction and whole blood.

The correlations between the metal(loid) concentrations in each matrix and the age of the subjects were also studied (Supplementary Table 3). In whole blood, there was a significant positive correlation of As and Pb with age (*r* = 0.367, *p* < 0.001 and *r* = 0.451, *p* < 0.001, respectively), and a significant negative correlation of Cu with age (*r* =  − 0.239, *p* = 0.006), with younger volunteers having higher concentrations, which was also observed in scalp hair (*r* =  − 0.337, *p* < 0.001). In fingernails, a significant positive correlation of Mn concentration with age (*r* = 0.315, *p* = 0.001) was observed.

Between metal(loid)s correlations in whole blood, scalp hair and fingernails are shown in Supplementary Tables 4–6, respectively. In whole blood, there were significant positive correlations between Mn/Fe (*r* = 0.191, *p* = 0.030), Fe/Zn (*r* = 0.283, *p* = 0.001), Cu/Zn (*r* = 0.180, *p* = 0.040) and As/Pb (*r* = 0.443, *p* < 0.001). Regarding to scalp hair, all of them were positive: Mn/Fe (*r* = 0.282, *p* = 0.002), Mn/Cd (*r* = 0.442, *p* < 0. 001), Mn/Pb (*r* = 0.348, *p* < 0.001), Fe/Cd (*r* = 0.288, *p* = 0.001), Cu/Cd (*r* = 0.189, *p* = 0.037), Cu/Pb (*r* = 0.345, *p* < 0.001) and Cd/Pb (*r* = 0.504, *p* < 0.001). Finally, the correlations in fingernails between Mn/Fe (*r* = 0.268, *p* = 0.004), Mn/Pb (*r* = 0.414, *p* < 0.001), Fe/Zn (*r* = 0.340, *p* < 0.001), Fe/Pb (*r* = 0.253, *p* = 0.013) and Cu/Pb (*r* = 0.211, *p* = 0.039) were also significant.

Between matrices correlations for detected metal(loid)s are shown in Supplementary Tables 7 (a-e). Regarding to Mn, no significant correlations between whole blood and any of the Mn fractions analysed in PM nor other biomarkers were observed; however, scalp hair Mn showed significant positive correlations with bioaccessible and total Mn in both fractions (coarse and fine), and fingernails Mn correlated well with all PM fractions analysed. Significant positive correlations were also observed for Fe between concentrations in scalp hair and fingernails (*r* = 0.228, *p* = 0.018) and between Pb concentrations in whole blood and scalp hair (*r* = 0.210, *p* = 0.020).

## Discussion

The discussion is first focused on Mn, because the study area is characterised by the presence of a ferromanganese alloy factory as the main source of metal(loid) emissions (Hernández-Pellón & Fernández-Olmo, 2019; Hernandez-Pellón et al., 2017), and due to the lack of consensus for the choice of a suitable biomarker for Mn, which is further difficulted by its role as a micronutrient (Aschner & Aschner, 2005; Hassani et al., 2016; Jursa et al., 2018). In addition, according to a previous study, this plant is practically the only source of airborne Mn in the study area (Otero-Pregigueiro et al., 2018).

In general, Mn levels in the studied markers are higher in the highly exposed group, with the exception of whole blood. This is corroborated when the correlation between Mn levels in these (bio)markers is analysed. Among the studied indicators of the Mn exposure, the PM-bound Mn concentration showed the highest differences between exposure groups, mainly for the bioaccessible fractions (see Figs. 2, 3, 4). Effect sizes and statistical significance were maintained after adjusting for potential confounders except for the non-bioaccessible fine fraction (see Table 4), highlighting the importance of the inhalation route of exposure to Mn from the ferromanganese industry emissions, in agreement with previous modelling and stationary sampling studies (Hernández-Pellón & Fernández-Olmo, 2019; Otero-Pregigueiro et al., 2018), with the improvement of accurate 24-h exposure monitoring in which each volunteer carried the personal sampler. Table 5 also confirmed this hypothesis, since the highest correlations between Mn levels and weighted distance were obtained for PM filters, with higher correlation coefficients for the bioaccessible fractions with respect to the non-bioaccessible fractions.

Although the measured Mn levels in biomarkers can result from the three routes of exposure, the results discussed above on PM-bound Mn concentrations and the importance of the bioaccessible fraction can explain the results obtained when scalp hair and fingernails are used. Thus, as shown in Fig. 4, we can consider scalp hair and mainly fingernails as potential biomarkers of long-term airborne Mn exposure. The most exposed population showed medians of 321.6 ng/g and 917.9 ng/g in scalp hair and fingernails, respectively, with these concentrations being notably lower for the less exposed population (132.7 ng/g and 331.3 ng/g, respectively), as evidenced in other studies (Coetzee et al., 2016; Haynes et al., 2015; Levin-Schwartz et al., 2021). These differences are again maintained after adjusting for potential confounders (see Table 4). The appropriateness of these biomarkers is also corroborated by the results of the calculation of between matrices correlations for Mn. We observed positive correlations with bioaccessible and total Mn in both fractions (coarse and fine) in the case of scalp hair, and with all PM fractions analysed in the case of fingernails.

Our results also suggest that whole blood is not a good biomarker of short-term airborne Mn exposure (see Fig. 3), being unable to differentiate between highly and moderately exposed groups (medians 9.16 µg/L vs. 9.18 µg/L, *p* = 0.865), even after adjusting for potential confounders. This is in agreement with previous studies reporting that Mn levels are tightly regulated in this matrix, with excess being quickly eliminated by the liver and excreted in bile and urine (Gurol et al., 2022). The correlation analysis also confirmed that whole blood cannot be used as biomarker of environmental exposure to Mn with epidemiological purposes, as it did not show significant correlations with any of the Mn fractions analysed in PM, nor with other biomarkers, nor with distance to the source.

With respect to Pb, Zn and Fe, their presence in the study area was also mainly attributed to the ferroalloy factory (Hernández-Pellón & Fernández-Olmo, 2019). This was supported by the significant positive correlations between Mn, Pb, Zn and Fe found in fingernails. However, other nearby emission sources such as a steel plant for Pb, Zn and Fe and non-exhaust road traffic for Zn and Fe cannot be ruled out. Regarding to Pb, blood has been considered as a reliable biomarker of exposure (Barbosa et al., 2005). However, although the levels of Pb in whole blood were higher in the highly exposed group (see Fig. 3), the difference was not statistically significant (*p* = 0.106). It should be noted that the steel plant is located only 3.5 km N from the ferroalloy factory, so its emissions can affect both the highly and the moderately exposed groups. Moreover, contradictory results were found for long-term biomarkers: a positive correlation with distance in scalp hair and negative in fingernails, both significant (see Table 5). Thus, while Fig. 4 depicts significant differences between groups in fingernails, with higher levels in the highly exposed group (103.3 vs. 82.8 ng/g, *p* = 0.025), but contrary to our hypothesis, Pb concentration in scalp hair was slightly higher in the moderately exposed group (*p* = 0.088). Although it is well known that blood and nails can be effective biomarkers of Pb exposure (Barbosa et al., 2005; Olympio et al., 2020), the contradictory results shown here for the three biomarkers studied need further research to elucidate (i) the suitability of these biomarkers to account for the exposure to airborne Pb, and (ii) the contribution of local emission sources other than ferroalloy smelting.

This study also confirmed that the actual levels of whole blood Pb are much lower than those of previous decades, even in industrial areas like Santander Bay, due to the strict regulations given worldwide. For example, RVs of 70 and 90 µg/L was derived by the Human Biomonitoring (HBM) Commission for women and men, respectively, in the period 1997–1999 (Schulz et al., 2011), much higher than the RV of 24.82 µg/L measured in this work. It also agrees with the report produced by the US Department of Health and Human Services (2018), with a P_95_ of 23.9 µg/L in 2015/2016. Other recent studies showed similar levels, such as those shown by Saravanabhavan et al. (2017) in a Canadian biomonitoring study, with a P_95_ of 33 µg/L, or by Ferreira et al. (2019) in an unexposed population in Brazil (P_95_ = 22.5 µg/L). In Spain, a former national biomonitoring study carried out between 2009 and 2010 (BIOAMBIENT) reported a P_95_ value of 56.8 µg/L (Cañas et al., 2014), evidencing this progressive decrease of Pb levels in blood.

With respect to Zn and Fe, a significant negative correlation with distance to the source was observed for Zn in scalp hair (*r* =  − 0.207, *p* = 0.035), but not in fingernails (*r* =  − 0.032, *p* = 0.754), while no correlation with distance in fingernails and even positive significant correlation in scalp hair was found for Fe (*r* = 0.269, *p* = 0.006). Again, the presence of other sources of these metals in the area makes it difficult to interpret these results. In addition, Zn and Fe are essential trace elements that can enter into the body by other routes.

Copper shows no significant differences as a function of exposure in whole blood and fingernails, although it does in scalp hair, but losing significance after adjusting for the selected confounders, mainly attributed to age differences (older people in the highly exposed group, as shown in Table 1, and negative correlation between scalp hair Cu and age, as shown in Supplementary Table 3). In any case, higher scalp hair Cu levels are measured in the moderately exposed population, in agreement with a positive significant correlation with distance (*r* = 0.311, *p* = 0.001), concluding that the origin of this metal is not due to the ferroalloys plant. According to the literature, airborne Cu originates mainly from non-exhaust emissions due to brake wear (Amato et al., 2010; Bäckström et al., 2003; Johansson et al., 2009). These results are consistent, as road traffic is similar or even higher in Santander area, where most of the moderately exposed group lives.

Finally, As exhibited important differences in whole blood (see Fig. 3), with the most exposed population showing statistically significant higher levels (4.24 µg/L vs. 2.81 µg/L, *p* = 0.002). This agrees with a significant negative correlation found with the distance to the factory (*r* =  − 0.239, *p* = 0.011). However, it seems that it does not bioaccumulate long-term in the body, remaining below the LOD in both scalp hair and fingernails. The origin of As in this area needs further investigation, since although it was measured in previous studies both in PM_10_ collected by stationary samplers (Hernández-Pellón & Fernández-Olmo, 2019) and in soil (Boente et al., 2020) near the ferromanganese plant, the levels of As in the bioaccessible and non-bioaccessible fractions of the personal filters collected in the present study were below the LOD, so other sources and routes of exposure are not ruled out.

The differences with respect to the sex of the participants are in line with those reported in previous studies (Bocca et al., 2011; Coelho et al., 2014; Saravanabhavan et al., 2017; Stojsavljević et al., 2019). Coelho et al. (2014) reported higher levels of Mn in toenails in females, in agreement with our results using fingernails. The higher whole blood Pb concentrations found in males are in accordance with those reported by Batáriová et al. (2006), Coelho et al. (2014), Schulz et al. (2011), Stojsavljević et al. (2019) and Zhang et al. (2015). Moreover, the higher whole blood Cu levels in females are in agreement with Bocca et al. (2011) and Zeng et al. (2019), and may be due to the fact that estrogen-induced ceruloplasmin synthesis in the liver, can lead to increased blood Cu levels in females (Prasad et al., 2014). On the other hand, no significant sex differences in whole blood Mn and As levels have been found, supported by Freire et al. (2015), Haynes et al. (2010), Nisse et al. (2017), Stojsavljević et al. (2019) and Zeng et al. (2019). However, contrary to us, Zeng et al. (2019) reported higher levels of Zn, Fe and As in the whole blood of males.

For males, significant higher levels of Mn in scalp hair were also observed, which is in agreement with Viana et al. (2014), although the significance they reported is limited. Nonetheless, other literature studies reported no significant differences in hair Mn levels according to sex (Haynes et al., 2010; Menezes-Filho et al., 2009; Riojas-Rodríguez et al., 2010).

The biomonitoring levels obtained in this study were also compared with the available literature. In the case of whole blood, our biomonitoring values were within the range or slightly below those reported in the literature with the exception of As; for this metal(loid), some outliers were measured, corresponding to participants living in the vicinity of the main source of metals in Maliaño. It is unusual to find As values in whole blood higher than 15 µg/L, although there have been previous studies reporting As levels similar to our outliers, such as those of Tratnik et al. (2019) (maximum As in women of 28.9 µg/L and in men of 22.4 µg/L), Freire et al. (2015), (maximum As in women of 26.28 µg/L and in men of 30.75 µg/L), Henríquez-Hernández et al. (2018) (maximum As of 29.12 µg/L in population aged 20–40 years in the Canary Islands (Spain)), and Kim et al. (2017) in South Korea (maximum As of 59.8 µg/L in women and 53.1 µg/L in men).

In the case of Mn, the median (9.76 µg/L) was slightly higher than that found in the USA from the Fourth National Report on Human Exposure to Environmental Chemicals, 9.52 µg/L in 2015/2016 from a sample of 4987 participants (US Department of Health & Human Services, 2018). The highest values of Mn in whole blood corresponded to participants living near the manganese alloy plant, but their maximum (26.76 µg/L) was clearly below some values reported near industrial sources of Mn: for example, Santos-Burgoa et al. (2001) reported a maximum of 88 µg/L near Mn ore mines in Mexico.

With respect to scalp hair, our values were in general well below those documented, except for Zn and Cu, which were within the range of other studies. This may be due to the fact that our cleaning protocol is much more thorough than most protocols used by other authors, leading to a complete removal of exogenous contamination of scalp hair by trace metals. The influence of pre-treatment steps on the levels of metals in hair has been discussed in the literature (Eastman et al., 2013). These differences were also observed in the case of fingernails, with the peculiarity that the literature studies available to compare our range of values were more limited.

Our ranges of concentrations in scalp hair and fingernails were in the same order of magnitude as those obtained by Butler et al. (2019), who used the same cleaning protocol previously reported by Eastman et al. (2013). For example, for Mn, Cu and Pb in scalp hair, they obtained medians of 0.08, 9.57 and 0.17 µg/g, respectively, compared to the medians obtained in this study of 0.19, 9.79 and 0.15 µg/g, respectively. However, studies in which a less thorough cleaning protocol was used showed much higher Mn levels in hair (e.g. 6.9–31.3 µg/g in Menezes-Filho et al. (2009), 12 µg/g in Mohmand et al. (2015), and 9.7 µg/g for males and 4.4 µg/g for females in Viana et al. (2014)). For nails, something similar occurred, as Butler et al. (2019) obtained medians for Mn, Cu and Pb of 0.19, 2.66 and 0.1 µg/g, respectively, while for the same metals our medians were 0.56, 3.63 and 0.1 µg/g, respectively.

## Conclusions

Among the studied indicators of Mn exposure, PM-bound Mn concentrations showed the largest differences between the highly exposed and moderately exposed, highlighting the importance of the inhalation route of exposure to Mn from emissions of the ferromanganese industry. The higher fingernails Mn levels of people living near the Mn source and the significant positive correlations between fingernails Mn and all PM fractions (bioaccessible/non-bioaccessible coarse and fine) confirm it as the best biomarker of long-term exposure to Mn, the main pollutant in Santander Bay according to WHO guidelines.

With respect to Pb, Zn and Fe, their presence in the study area was also mainly attributed to the ferroalloy factory; however, other nearby emission sources cannot be ruled out, such as a steel plant for Pb, Zn and Fe and non-exhaust road traffic for Zn and Fe; moreover, Zn and Fe are essential trace elements that can enter the organism by other routes. The contradictory results shown here for the three studied biomarkers, mainly for Pb, indicate the difficulties in interpreting the results when different environmental metal(loid) sources and routes of exposure to them may occur. Finally, this study also confirmed that current whole blood Pb levels, even in an urban-industrial mixed area, are much lower than in previous decades.

## Supplementary Information

Below is the link to the electronic supplementary material.

Supplementary file1 (DOCX 39 kb)
