# Supplementary material for: Short- and long-term exposure to trace metal(loid)s from the production of ferromanganese alloys by personal sampling and biomarkers
Source: Environ Geochem Health. 2022 Feb 22;44(12):4595–618. doi: 10.1007/s10653-022-01218-8 (PMC8860625; doi:10.1007/s10653-022-01218-8)
Supplement: Supplementary file 1 — Supplementary file1 (DOCX 39 kb) [file 10653_2022_1218_MOESM1_ESM.docx]

Short- and long-term exposure to trace metal(loid)s from the production of ferromanganese alloys by personal sampling and biomarkers

B. Markiv^1,*^, L. Ruiz-Azcona^2^, A. Expósito^1^, M. Santibáñez^2^, I. Fernández-Olmo^1^

^1^Department of Chemical and Biomolecular Engineering, Universidad de Cantabria, Spain

^2^Department of Nursing, Universidad de Cantabria, Spain.

^*^Corresponding author. Tel: +34 942 201579, E-mail: [markivb@unican.es](mailto:markivb@unican.es)

**Supplementary Table 1.** Detection limits and quantification limits for the studied metal(loid)s in whole blood, scalp hair, fingernails, and PM filters.

| Metal(loid) | Whole blood (µg/L) | | Scalp hair (ng/g) | | Fingernails (ng/g) | | Coarse, bioaccessible (ng/m^3^) | | | Coarse, non-bioaccessible (ng/m^3^) | | Fine, bioaccessible (ng/m^3^) | | Fine, non-bioaccessible (ng/m^3^) | |
| --- | --- | --- | --- | --- | --- | --- | --- | --- | --- | --- | --- | --- | --- | --- | --- |
|  | LOD | LOQ | LOD | LOQ | LOD | LOQ | | LOD | LOQ | LOD | LOQ | LOD | LOQ | LOD | LOQ |
| Mn | 0.74 | 3.27 | 3.37-115.86 | 14.91-512.65 | 9.76-89.23 | 43.19-394.82 | | 0.76 | 3.36 | 2.52 | 11.15 | 0.59 | 2.61 | 0.99 | 4.38 |
| Fe | 8.81 | 38.98 | 112.62-3870.08 | 498.32-17124.25 | 326.08-2981.19 | 1442.83-13191.11 | | 14.39 | 63.67 | 64 | 283.19 | 31.42 | 139.03 | 36.56 | 161.77 |
| Cu | 22.89 | 101.28 | 5.85-200.86 | 25.88-888.76 | 16.92-154.68 | 74.87-684.42 | | 0.86 | 3.81 | 15.15 | 67.04 | 3.48 | 15.40 | 6.07 | 26.86 |
| Zn | 9.97 | 44.12 | 21.49-738.34 | 95.09-3266.99 | 62.20-568.70 | 275.22-2516.37 | | 58.33 | 258.10 | 41.03 | 181.55 | 13 | 57.52 | 17.37 | 76.86 |
| As | 0.10 | 0.44 | 1.56-53.70 | 6.90-237.61 | 4.53-41.37 | 20.04-183.05 | | 0.04 | 0.18 | 0.07 | 0.31 | 0.24 | 1.06 | 0.06 | 0.27 |
| Cd | - | - | 0.71-24.53 | 3.14-108.54 | 2.07-18.88 | 9.16-83.54 | | 0.11 | 0.49 | 0.32 | 1.42 | 0.27 | 1.19 | 0.03 | 0.13 |
| Pb | 1.48 | 6.55 | 0.85-29.12 | 3.76-128.85 | 2.46-22.49 | 10.88-99.51 | | 5.74 | 25.40 | 1.84 | 8.14 | 0.42 | 1.86 | 0.73 | 3.23 |

**Supplementary Table 2.** Recoveries obtained on whole blood and hair reference materials, Seronorm™ Trace Elements Whole Blood L-1 and ERM®- DB001

| Metal | Certified whole blood value (µg/L) | Recovery (%) | Certified hair value (ng/g) | Recovery (%) |
| --- | --- | --- | --- | --- |
| Mn | 18.4 | 109.5 | 442* | 144.2 |
| Fe | 334000* | 100.5 | 22600* | 98.1 |
| Cu | 640 | 93.5 | 33000 | 109.2 |
| Zn | 4300 | 103.3 | 209000 | 93.1 |
| As | 2.4 | 112.1 | 44 | 116.6 |
| Cd | 0.28 | ** | 125 | 108.6 |
| Pb | 9.9 | 97.3 | 2140 | 100.6 |

*Informative values

**Below LOD

**Supplementary Table 3.** Spearman´s correlations between concentrations of all employed (bio)markers and age of volunteers, divided into two groups according to distance weighting: short-term and long-term.

|  | **R** | **p-value** |
| --- | --- | --- |
| **Short-term (bio)markers** |  |  |
| Mn coarse fraction bioaccessible (ng/m^3^) | 0.102 | 0.248 |
| Mn coarse fraction non-bioaccessible (ng/m^3^) | 0.080 | 0.364 |
| Mn coarse fraction total (ng/m^3^) | 0.108 | 0.223 |
| Mn fine fraction bioaccessible (ng/m^3^) | -0.069 | 0.439 |
| Mn fine fraction non-bioaccessible (ng/m^3^) | -0.001 | 0.988 |
| Mn fine fraction total (ng/m^3^) | -0.040 | 0.651 |
| Mn total fraction (PM_10_) (ng/m^3^) | 0.027 | 0.758 |
| Fe coarse fraction bioaccessible (ng/m^3^) | 0.070 | 0.431 |
| Fe fine fraction non-bioaccessible (ng/m^3^) | 0.037 | 0.678 |
| Pb fine fraction bioaccessible (ng/m^3^) | -0.062 | 0.481 |
| Whole blood Mn (µg/L) | -0.158 | 0.073 |
| Whole blood Fe (µg/L) | -0.025 | 0.777 |
| Whole blood Cu (µg/L) | -0.239 | 0.006 |
| Whole blood Zn (µg/L) | 0.049 | 0.584 |
| Whole blood As (µg/L) | 0.367 | <0.001 |
| Whole blood Pb (µg/L) | 0.451 | <0.001 |
| **Long-term biomarkers** |  |  |
| Scalp hair Mn (ng/g) | -0.077 | 0.398 |
| Scalp hair Fe (ng/g) | -0.112 | 0.219 |
| Scalp hair Cu (ng/g) | -0.337 | <0.001 |
| Scalp hair Zn (ng/g) | -0.103 | 0.261 |
| Scalp hair Cd (ng/g) | -0.070 | 0.443 |
| Scalp hair Pb (ng/g) | -0.059 | 0.520 |
| Fingernails Mn (ng/g) | 0.315 | 0.001 |
| Fingernails Fe (ng/g) | -0.013 | 0.894 |
| Fingernails Cu (ng/g) | -0.094 | 0.361 |
| Fingernails Zn (ng/g) | -0.048 | 0.609 |
| Fingernails Pb (ng/g) | 0.020 | 0.845 |

**Supplementary Table 4.** Spearman´s correlation coefficients (p-value) among metal(loid)s in whole blood.

|  | Mn blood (µg/L) | Fe blood (µg/L) | Cu blood (µg/L) | Zn blood (µg/L) | As blood (µg/L) | Pb blood (µg/L) |
| --- | --- | --- | --- | --- | --- | --- |
| Mn blood (µg/L) | 1 |  |  |  |  |  |
| Fe blood (µg/L) | **0,191* (0.030)** | 1 |  |  |  |  |
| Cu blood (µg/L) | 0.104 (0.240) | -0.008 (0.926) | 1 |  |  |  |
| Zn blood (µg/L) | 0.163 (0.064) | **0,283** (0.001)** | **0,180* (0.040)** | 1 |  |  |
| As blood (µg/L) | -0.115 (0.192) | -0.057 (0.519) | 0.093 (0.293) | 0.071 (0.421) | 1 |  |
| Pb blood (µg/L) | 0.027 (0.759) | 0.127 (0.149) | -0.036 (0.681) | 0.101 (0.255) | **0,443** (0.000)** | 1 |

**Supplementary Table 5.** Spearman´s correlation coefficients (p-value) among metal(loid)s in scalp hair.

|  | Mn hair (ng/g) | Fe hair (ng/g) | Cu hair (ng/g) | Zn hair (ng/g) | Cd hair (ng/g) | Pb hair (ng/g) |
| --- | --- | --- | --- | --- | --- | --- |
| Mn hair (ng/g) | 1 |  |  |  |  |  |
| Fe hair (ng/g) | **0.282** (0.002)** | 1 |  |  |  |  |
| Cu hair (ng/g) | 0.086 (0.348) | 0.146 (0.109) | 1 |  |  |  |
| Zn hair (ng/g) | 0.038 (0.680) | -0.157 (0.085) | -0.061 (0.504) | 1 |  |  |
| Cd hair (ng/g) | **0.442** (0.000)** | **0.288** (0.001)** | **0.189** (0.037)** | 0.124 (0.175) | 1 |  |
| Pb hair (ng/g) | **0.348** (0.000)** | 0.151 (0.098) | **0.345** (0.000)** | 0.115 (0.209) | **0.504** (0.000)** | 1 |

**Supplementary Table 6.** Spearman´s correlation coefficients (p-value) among metal(loid)s in fingernails.

|  | Mn fingernails (ng/g) | Fe fingernails (ng/g) | Cu fingernails (ng/g) | Zn fingernails (ng/g) | Pb fingernails (ng/g) |
| --- | --- | --- | --- | --- | --- |
| Mn fingernails (ng/g) | 1 |  |  |  |  |
| Fe fingernails (ng/g) | **0.268** (0.004)** | 1 |  |  |  |
| Cu fingernails (ng/g) | 0.093 (0.369) | 0.136 (0.188) | 1 |  |  |
| Zn fingernails (ng/g) | 0.090 (0.339) | **0.340** (0.000)** | 0.160 (0.119) | 1 |  |
| Pb fingernails (ng/g) | **0.414** (0.000)** | **0.253* (0.013)** | **0.211* (0.039)** | 0.193 (0.060) | 1 |

**Supplementary Table 7.** Spearman´s correlation coefficients (p-value) among metal(loid)s in different matrices: **a)** Mn, **b)** Fe, **c)** Cu, **d)** Zn **e)** Pb.

**a)** Manganese

|  | Mn Blood | Mn Hair | Mn Fingernails | Mn Coarse Fraction Bioaccessible | Mn Coarse Fraction Non-Bioaccessible | Mn Coarse Fraction Total | Mn Fine Fraction Non-Bioaccessible | Mn Fine Fraction Bioaccessible | Mn Fine Fraction Total | Mn TotalFraction (PM_10_) |
| --- | --- | --- | --- | --- | --- | --- | --- | --- | --- | --- |
| Mn Blood | 1 |  |  |  |  |  |  |  |  |  |
| Mn Hair | 0.143  (0.116) | 1 |  |  |  |  |  |  |  |  |
| Mn Fingernails | -0.179  (0.054) | 0.169  (0.080) | 1 |  |  |  |  |  |  |  |
| Mn Coarse Fraction Bioaccessible | -0.060  (0.497) | **0.224***  **(0.013)** | **0.439****  **(<0.001)** | 1 |  |  |  |  |  |  |
| Mn Coarse Fraction Non-Bioaccessible | 0.017  (0.848) | 0.175  (0.054) | **0.371****  **(<0.001)** | **0.750****  **(<0.001)** | 1 |  |  |  |  |  |
| Mn Coarse Fraction Total | -0.056  (0.526) | **0.207***  **(0.022)** | **0.434****  **(<0.001)** | **0.983****  **(<0.001)** | **0.831****  **(<0.001)** | 1 |  |  |  |  |
| Mn Fine Fraction Non-Bioaccessible | -0.127  (0.151) | 0.160  (0.079) | **0.255****  **(0.006)** | **0.631****  **(<0.001)** | **0.494****  **(<0.001)** | **0.621****  **(<0.001)** | 1 |  |  |  |
| Mn Fine Fraction Bioaccessible | -0.044  (0.617) | **0.191***  **(0.035)** | **0.235***  **(0.011)** | **0.826****  **(<0.001)** | **0.596****  **(<0.001)** | **0.794****  **(<0.001)** | **0.703****  **(<0.001)** | 1 |  |  |
| Mn Fine Fraction Total | -0.073  (0.408) | **0.192***  **(0.034)** | **0.258****  **(0.005)** | **0.813****  **(<0.001)** | **0.608****  **(<0.001)** | **0.789****  **(<0.001)** | **0.829****  **(<0.001)** | **0.969****  **(<0.001)** | 1 |  |
| Mn Total Fraction (PM_10_) | -0.069  (0.437) | **0.202***  **(0.026)** | **0.356****  **(0.000)** | **0.920****  **(<0.001)** | **0.737****  **(<0.001)** | **0.916****  **(<0.001)** | **0.776****  **(<0.001)** | **0.943****  **(<0.001)** | **0.957****  **(<0.001)** | 1 |

**b)** Iron

|  | Fe Blood | Fe Hair | Fe Fingernails | Fe Coarse Fraction Bioaccessible | Fe Fine Fraction Non-Bioaccessible |
| --- | --- | --- | --- | --- | --- |
| Fe Blood | 1 |  |  |  |  |
| Fe Hair | -0.018 (0848) | 1 |  |  |  |
| Fe Fingernails | 0.106 (0.256) | **0.228* (0.018)** | 1 |  |  |
| Fe Coarse Fraction Bioaccessible | 0.071 (0.420) | 0.035 (0.699) | 0.140 (0.133) | 1 |  |
| Fe Fine Fraction Non-Bioaccessible | -0.033 (0.712) | -0.106 (0.243) | -0.005 (0.961) | 0.161 (0.067) | 1 |

**c)** Copper

|  | Cu Blood | Cu Hair | Cu Fingernails |
| --- | --- | --- | --- |
| Cu Blood | 1 |  |  |
| Cu Hair | 0.125 (0.170) | 1 |  |
| Cu Fingernails | -0.187 (0.068) | -0.070 (0.510) | 1 |

**d)** Zinc

|  | Zn Blood | Zn Hair | Zn Fingernails |
| --- | --- | --- | --- |
| Zn Blood | 1 |  |  |
| Zn Hair | -0.092 (0.312) | 1 |  |
| Zn Fingernails | 0.019 (0.843) | -0.102 (0.294) | 1 |

**e)** Lead

|  | Pb Blood | Pb Hair | Pb Fingernails | Pb Fine Fraction Bioaccessible |
| --- | --- | --- | --- | --- |
| Pb Blood | 1 |  |  |  |
| Pb Hair | **0.210* (0.020)** | 1 |  |  |
| Pb Fingernails | 0.019 (0.855) | 0.064 (0.548) | 1 |  |
| Pb Fine Fraction Bioaccessible | 0.088 (0.321) | -0.028 (0.763) | -0.036 (0.704) | 1 |
